# Predictive value of molecular subtyping in NMIBC by RT-qPCR of ERBB2, ESR1, PGR and MKI67 from formalin fixed TUR biopsies

**DOI:** 10.18632/oncotarget.18804

**Published:** 2017-06-28

**Authors:** Johannes Breyer, Ralph Markus Wirtz, Wolfgang Otto, Mark Laible, Kornelia Schlombs, Philipp Erben, Maximilian Christian Kriegmair, Robert Stoehr, Sebastian Eidt, Stefan Denzinger, Maximilian Burger, Arndt Hartmann

**Affiliations:** ^1^ Department of Urology, University of Regensburg, Regensburg, Germany; ^2^ STRATIFYER Molecular Pathology GmbH, Cologne, Germany; ^3^ Institute of Pathology at The St Elisabeth Hospital Köln-Hohenlind, Cologne, Germany; ^4^ BioNTech Diagnostics GmbH, Mainz, Germany; ^5^ Department of Urology, University Medical Centre Mannheim, Medical Faculty Mannheim, University of Heidelberg, Mannheim, Germany; ^6^ Institute of Pathology, University of Erlangen-Nuremberg, Erlangen, Germany

**Keywords:** molecular subtypes, non-muscle-invasive bladder cancer, prognosis, RT-qPCR, mRNA

## Abstract

Expression of ESR1, PGR, HER2 and Ki67 is important for risk stratification and therapy in breast cancer. Hormone receptor expression can also be found in MIBC, reflecting luminal and basal subtypes of breast cancer. Thus the purpose was to investigate on the mRNA expression of the aforementioned markers and their prognostic value in pT1 bladder cancer.

Retrospective analysis of clinical data and Formalin-Fixed Paraffin-Embedded tissues (FFPE) of patients with stage pT1 NMIBC who underwent transurethral resection of the bladder was performed. mRNA expression was measured by single step RT-qPCR. Relative gene expression was determined by normalization to two housekeeping genes (CALM2, B2M) using the 40-ΔΔCT method. Correlation of mRNA expression with outcome was assessed using Kaplan-Meier analysis and multivariate Cox regression analysis.

From overall 302 patients, 255 samples could be analyzed with valid measurements. Subtype distribution was Luminal-A in 11.4%, Luminal-B in 38.8%, triple negative in 36.9% and ERBB2 in 12.9%, respectively. Kaplan-Meier analysis revealed molecular subtyping being statistical significant for RFS (p=0.0408) and PFS (p=0.0039). Luminal-A patients did have the best RFS and PFS. Multivariate analysis revealed molecular subtyping to be significant for PFS (L-R Chi^2^ of 11.89, p=0.0078). Elevated expression of HER2 was statistically significant for PFS (p=0.0025) and discriminated among G3 tumors a high risk group (60% PFS) from a low risk risk group (90% PFS) after 5 year follow-up (p<0.001).

Expression of ESR1, PGR and HER2 has predictive value in stage pT1 NMIBC and reveals potential therapeutic targets.

## INTRODUCTION

Bladder cancer is the fifth most common malignancy worldwide with an estimated 386.300 new cases annually [1]. Approximately 75% of the patients are diagnosed with non-muscle-invasive bladder cancer (NMIBC) [2]. After transurethral resection 50-70% of the patients have disease recurrence and up to one third of the patients develop disease progression to muscle-invasive disease [3]. Patients with NMIBC are monitored with cystoscopies over many years, which impose heavy costs to society and bladder cancer carries the highest cost among cancers per patient from diagnosis to death [4]. Therefore molecular markers for stratifying patient treatment and application of novel therapeutic drugs are highly appreciated especially for stage pT1 bladder cancer.

More recently, molecular inter-tumor heterogeneity of bladder cancer has been investigated leading to the identification of distinct molecular classes beyond histopathological classification resembling molecular features of the luminal and basal breast cancer subtypes with similar differences in clinical outcomes [5, 6, 7, 8, 9]. Most studies have focused primarily on muscle-invasive bladder cancers (MIBC). Combined analysis of all stages of bladder cancer has identified five major molecular groups (Urobasal A, Genomically Unstable, Infiltrated, Urobasal B, and SCC-like) [9]. MIBC has been shown to be of mainly basal- and luminal- cell origin [5, 6, 7], reflecting molecular classification of breast cancer. In addition, a p53-like subclass has been identified that determines MIBC patients resistant to cisplatin-based chemotherapy [6]. However, this p53 subclass is characterized by low proliferative status and elevated hormone receptor expression and thereby can be interpreted as the Luminal-A like subtype similar to breast cancer. Classifying patients according to molecular subgroups may provide novel diagnostic tools to identify patients responsive to targeted treatment [10]. For muscle-invasive disease, the direct comparison of the breast cancer subtypes and bladder cancer subtypes has been shown [7] and the four targeted genes ESR1, PGR, ERBB2 and MKI67 have been shown to be of diverse expression in bladder cancer [7, 14].

Unlike the situation in breast cancer, diagnosis and treatment in bladder cancer currently still depends on histopathologic evaluations not including IHC based molecular subtyping though certain parameters such as HER2 mRNA expression could be used to distinguish luminal from basal type bladder cancer [5].

In view of numerous technical limitations of the IHC based assessment, we used an established and certified RT-qPCR based assessment that is in clinical routine use for breast cancer (MammaTyper) to enable objective quantitation of mRNA expression of ESR1, PGR, ERBB2 and MKI67 in stage pT1 NMIBC with superior sensitivity and prognostic value. Furthermore, we aimed to assess, the distinction of molecular subtypes in NMIBC similar to breast cancer and to evaluate their predictive potential.

## RESULTS

### Patient population

**Figure 1:**
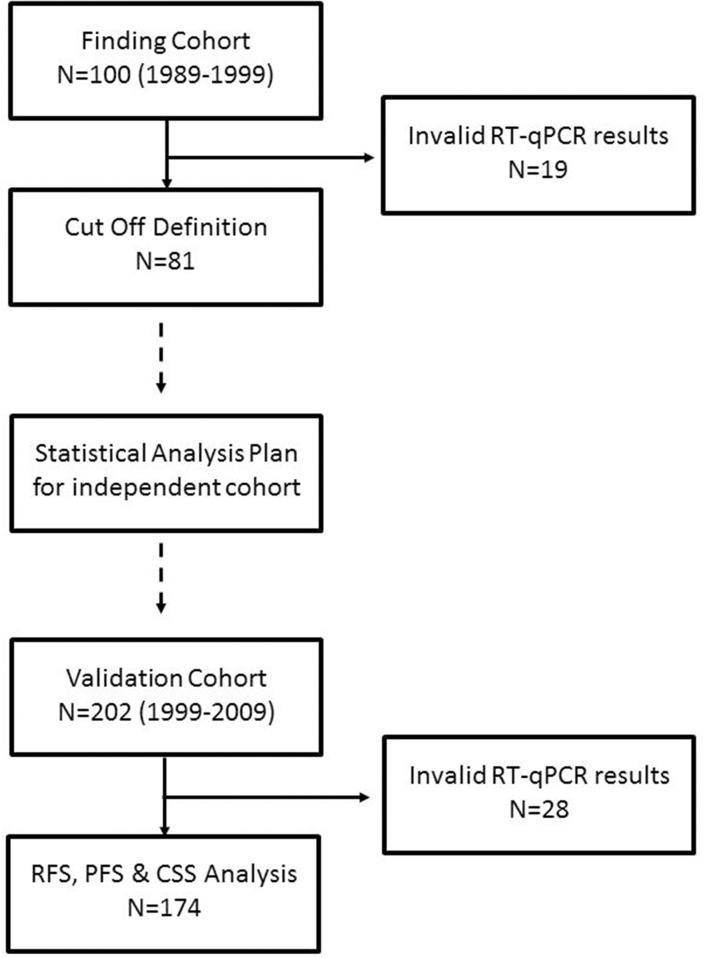
Remark diagram

**Figure 2:**
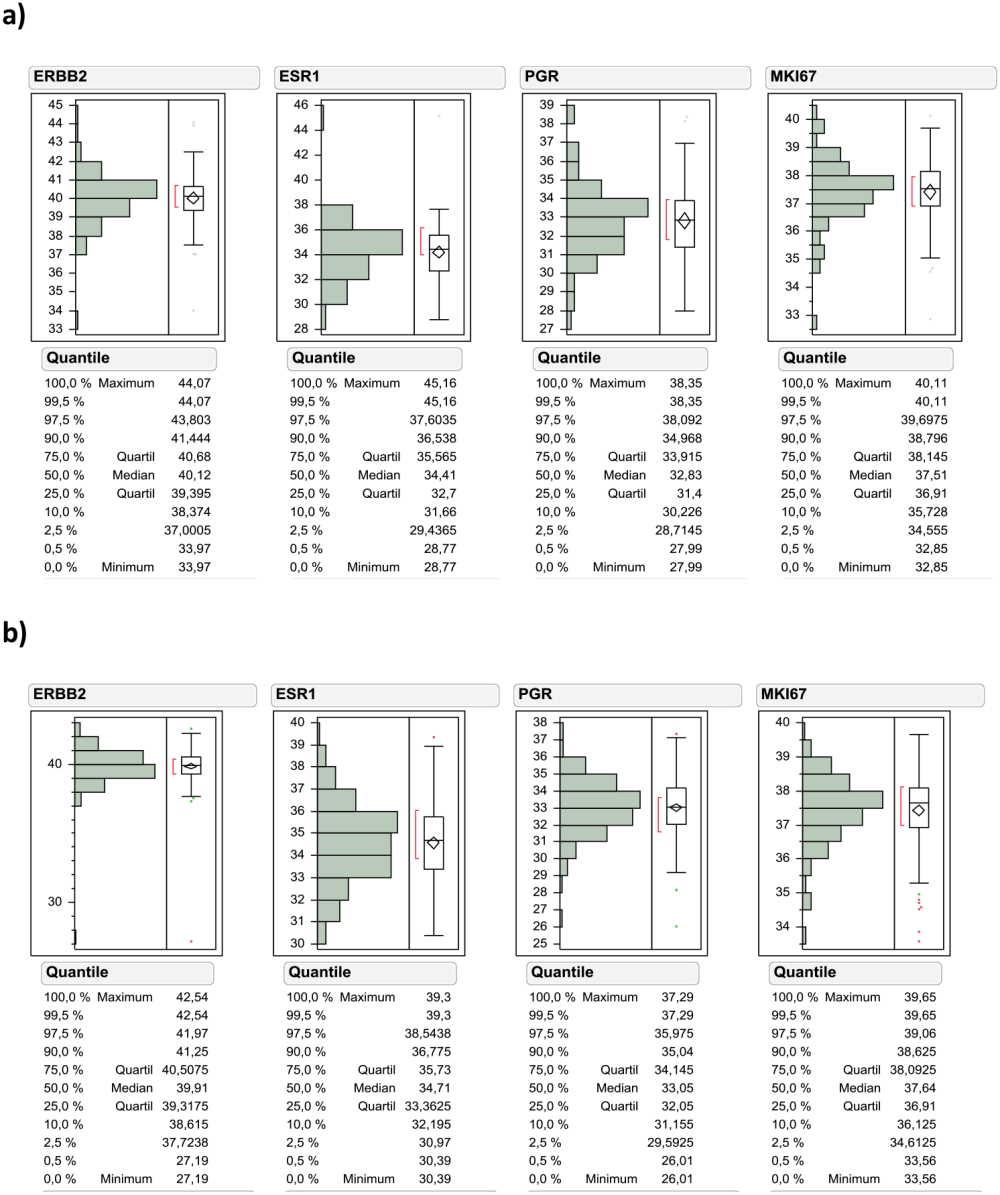
Data distribution of ERBB2, ESR1, PGR and MKI67 mRNA levels in the finding cohort **(a)** and validation cohort **(b)**.

The total study cohort consisted of 302 NMIBC tumor samples staged pT1 after central pathological review, which has been split into a finding cohort (n=100) and validation cohort (n=202). According to predefined criteria 255 samples out of 302 tumor samples (84.4%) could be analyzed with valid measurements for all four biomarkers (Figure 1). The invalid measurements in 47 samples were due to insufficient RNA input according to the pre-specified criteria of the MammaTyper kit and described in the instruction for use of the IVD kit. Tumor samples from both cohorts were collected at a single institution. The finding cohort consisted of tumor samples from 1989-1999, the validation cohort from 2000-2009. Therefore the median follow-up time was shorter for the validation cohort compared to the finding cohort (33.5 vs. 62 months). The clinicopathological parameters in both cohorts were comparable (Table 1). The median mRNA expression of ERBB2, ESR1, PGR and MKI67 was balanced between the two cohorts, while there were slightly more ultrahigh MKI67 expressing tumors in the finding cohort (Figure 2).

**Table 1:** Characteristics of patients in finding and evaluation cohorts without statistical significant differences of relevant clinicopathologic aspects

Patient characteristics	Finding cohort n (%)	Evaluation cohort n (%)	p value
Total patient number included	81	174	
age ≤75 years	56 (69.1)	110 (63.2)	0.218
male gender	63 (77.8)	138 (79.3)	0.450
associated CIS	15 (18.5)	50 (28.7)	0.054
multifocal tumor	13 (16.0)	39 (22.4)	0.157
tumor size >3cm	47 (58.0)	102 (58.6)	0.517
WHO grading 1973 G2 WHO grading 1973 G3	20 (24.7) 61 (75.3)	50 (28.7) 124 (71.3)	0.303
WHO grading 2004 low grade WHO grading 2004 high grade	0 81 (100)	5 (2.9) 169 (97.1)	0.145
recurrence rate (tumors ≤ stage pT1)	23 (28.4)	48 (27.6)	0.503
progression rate	12 (14.8)	30 (17.2)	0.386
cancer-specific mortality	9 (11.1)	15 (8.6)	0.110

CIS: carcinoma in situ, WHO: World Health Organization.

### Prognostic value of molecular subtyping

**Figure 3:**
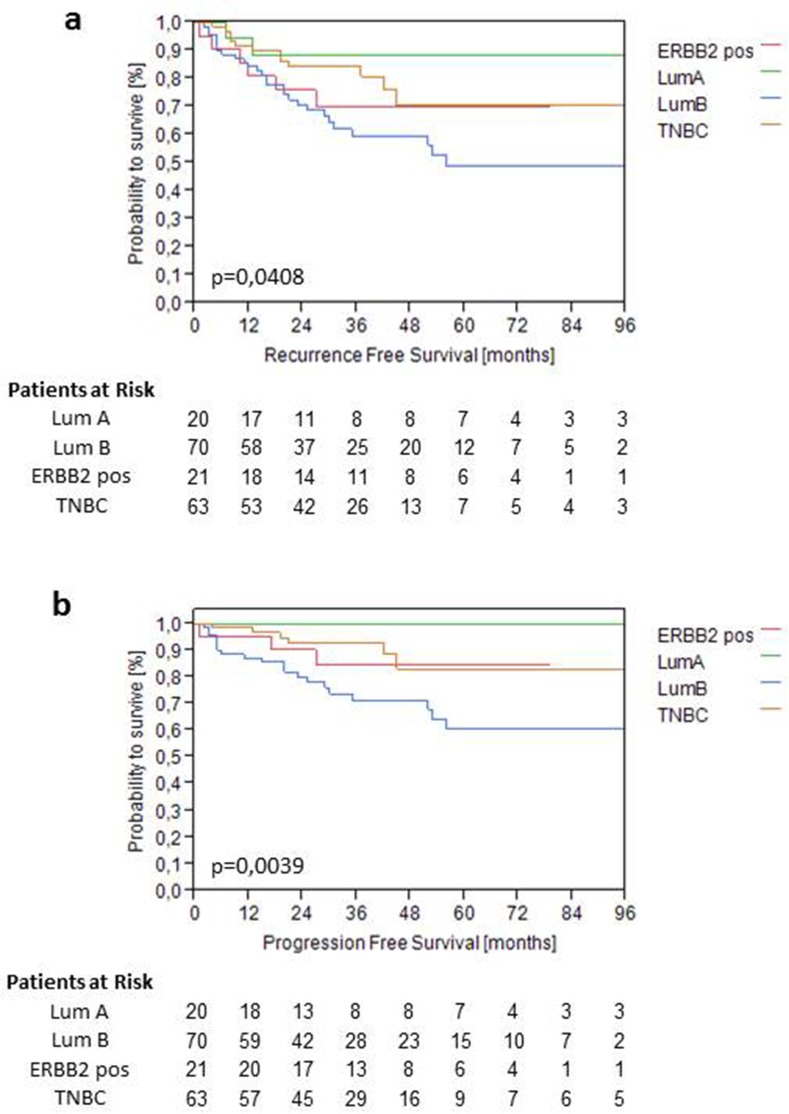
Kaplan-Meier analysis of RFS (a) and PFS (b) based on predefined molecular subtypes in the validation cohort

Hormone receptor and HER2 expression was as following: HER2 negative in 91.4%, ESR1 negative in 70.3%, PGR negative in 22.9% and Ki67 low in 27.4% of the cases. Classifying pT1 UBC tumor samples (all technically valid samples; n=255) by a proprietary algorithm results in the following subtype distribution: 36.9% TNBC, 38.8% Luminal-B, 11.4% Luminal-A and 12.9% ERBB2 positive tumors (Table 2). Within the validation cohort the molecular subtyping was significant for RFS (p=0.0408) and PFS (p=0.0039) as determined by Kaplan-Meier survival analysis (Figure 3). For CSS only a trend could be found (p=0.1961). Luminal-A tumors had the best RFS and PFS with no cancer specific death in the Luminal-A subtype. Luminal-B tumors had a worse prognosis than ERBB2 positive and TNBC.

**Table 2:** Simplified marker based subtypes used in this study and distribution

HER2/ERBB2	ER/ESR1	PR/PGR	Ki-67/MKI67	Subtype definition	%
pos	pos	pos	pos		
pos	pos	pos	neg		
pos	pos	neg	pos		
pos	pos	neg	neg	HER2 positive	12.9
pos	neg	pos	pos		
pos	neg	pos	neg		
pos	neg	neg	pos		
pos	neg	neg	neg		
neg	neg	pos	neg	Luminal-A-like	11.4
neg	pos	pos	neg		
neg	pos	neg	pos		
neg	pos	neg	neg	Luminal-B-like	38.8
neg	neg	pos	pos		
neg	pos	pos	pos		
neg	neg	neg	pos	Triple negative	36.9
neg	neg	neg	neg		

Multivariate Cox-regression analysis adjusted for gender, associated Cis, tumor size, focality, WHO 1973 grading and tumor subtype revealed that molecular subtyping retained its prognostic significance for PFS (L-R Chi^2^ 11.89 and p=0.0078), while all clinical variables lost their significance in the validation cohort (Table 3b). The relative risk for progression was particularly low for Luminal-A tumors compared to Luminal-B (HR 1.3xe^−6^, p=0.0062). Even after correction for clinical variables the Hazard ratio was particularly high for Luminal-B also when comparing to TNBC (HR 2.98, p=0.0141). For RFS the molecular subtyping overall resulted in trended to be significant after adjustment for the aforementioned parameters (L-R Chi^2^ 6.59 and p=0.0861), while all clinical variables did not retain any prognostic significance (Table 3a). When comparing Luminal-A to Luminal-B RFS was significant (HR 0.27, p=0.0351).

**Table 3:** Multivariate Cox regression analysis of clinic-pathological parameters and BladderTyper subtype regarding recurrence-free (a) and progression-free (b) survival in validation cohort (n=174)

a.	HR (CI 95%)	L-R Chi^2^		p-value	
**Gender** *female vs. male*	0.63 (0.27 - 1.30)	1.46		0.2277	
**Associated Cis** *yes vs. no*	1.31 (0.68 - 2.44)	0.68		0.4109	
**Tumor size** *>3cm vs. ≤3cm*	1.36 (0.73 - 2.64)	0.93		0.3352	
**Focality** *multifocality vs. unifocality*	0.87 (0.34 - 2.64)	0.12		0.7266	
**WHO Grading 1973** *G3 vs. G2*	1.01 (0.49 - 2.26)	0.00		0.9733	
**BladderTyper** *Subtype*		6.59		0.0861	
*LumA vs LumB*	0.27 (0.04 - 0.92)			**0.0351**	
*LumB vs TNBC*	1.96 (0.99 - 4.07)			0.0510	
*LumB vs ERBB2 pos*	1.44 (0.62 - 3.91)			0.4123	
**b.**	**HR (CI 95%)**		**L-R Chi^2^**	**p-value**
**Gender** *female vs. male*	0.63 (0.21 – 1.64)		0.93	0.3350
**Associated Cis** *yes vs. no*	1.42 (0.64 - 3.04)		0.80	0.3721
**Tumor size** *>3cm vs. ≤3cm*	1.37 (0.63 - 3.23)		0.59	0.4433
**Focality** *multifocality vs. unifocality*	0.85 (0.24 - 2.26)		0.09	0.7590
**WHO Grading 1973** *G3 vs. G2*	2.37 (0.78 - 10.22)		2.25	0.1336
**BladderTyper** *Subtype*			11.89	**0.0078**
*LumA vs LumB*	1.3xe^−6^ (0.00 - 0.48)			**0.0062**
*LumB vs TNBC*	2.98 (1.23 - 8.37)			**0.0141**
*LumB vs ERBB2 pos*	2.15 (0.73 - 9.14)			0.1780

Reference in italics, p-values <0.05 are indicated in bold; CI: confidence interval, Cis: carcinoma *in situ*; HR: hazard ratio, WHO: World Health Organization.

### Prognostic and predictive value of ERBB2 mRNA expression

**Figure 4:**
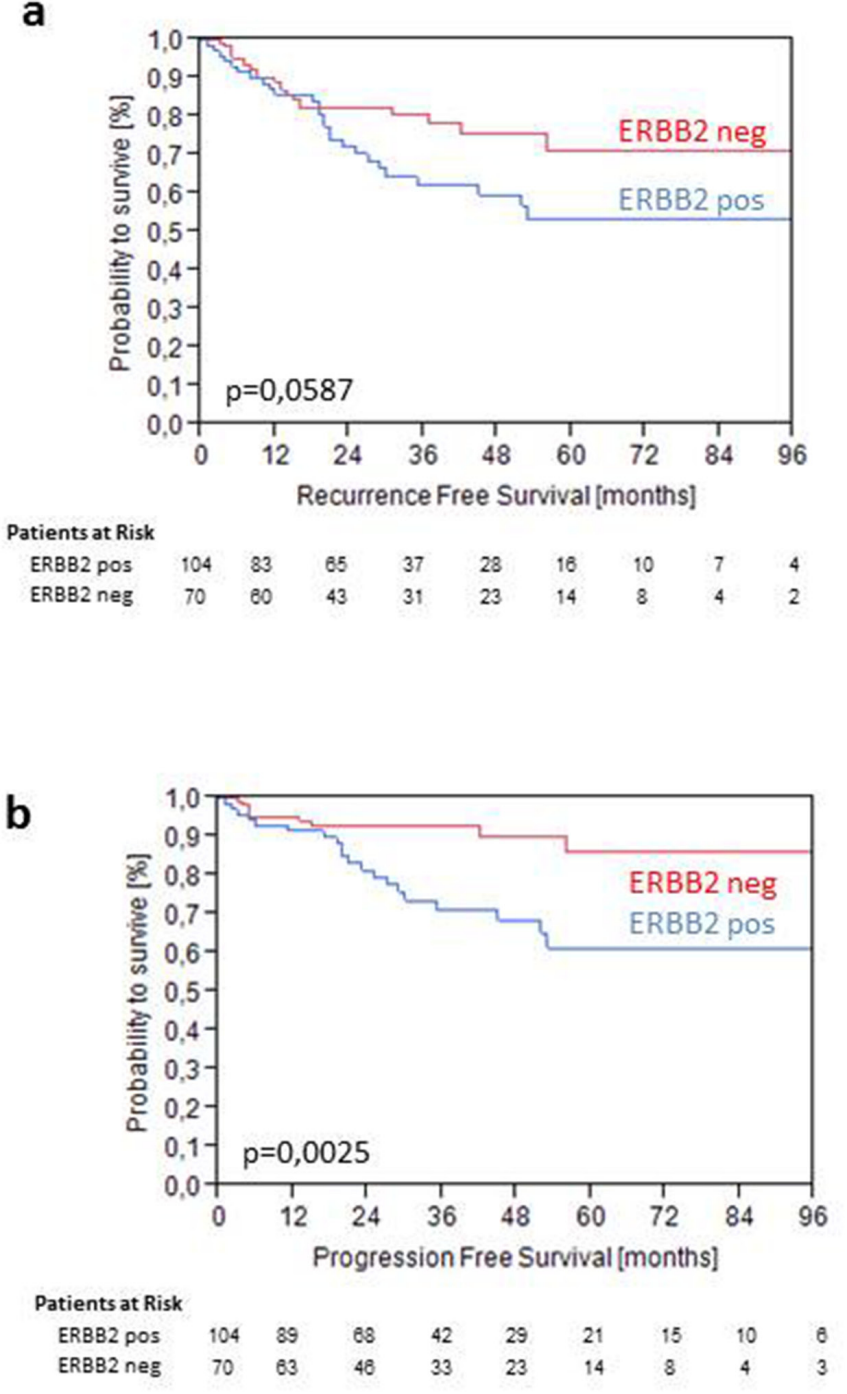
Kaplan-Meier analysis for RFS **(a)** and PFS **(b)** stratified by intermediate ERBB2 mRNA levels at 40.1 in the validation cohort.

**Figure 5:**
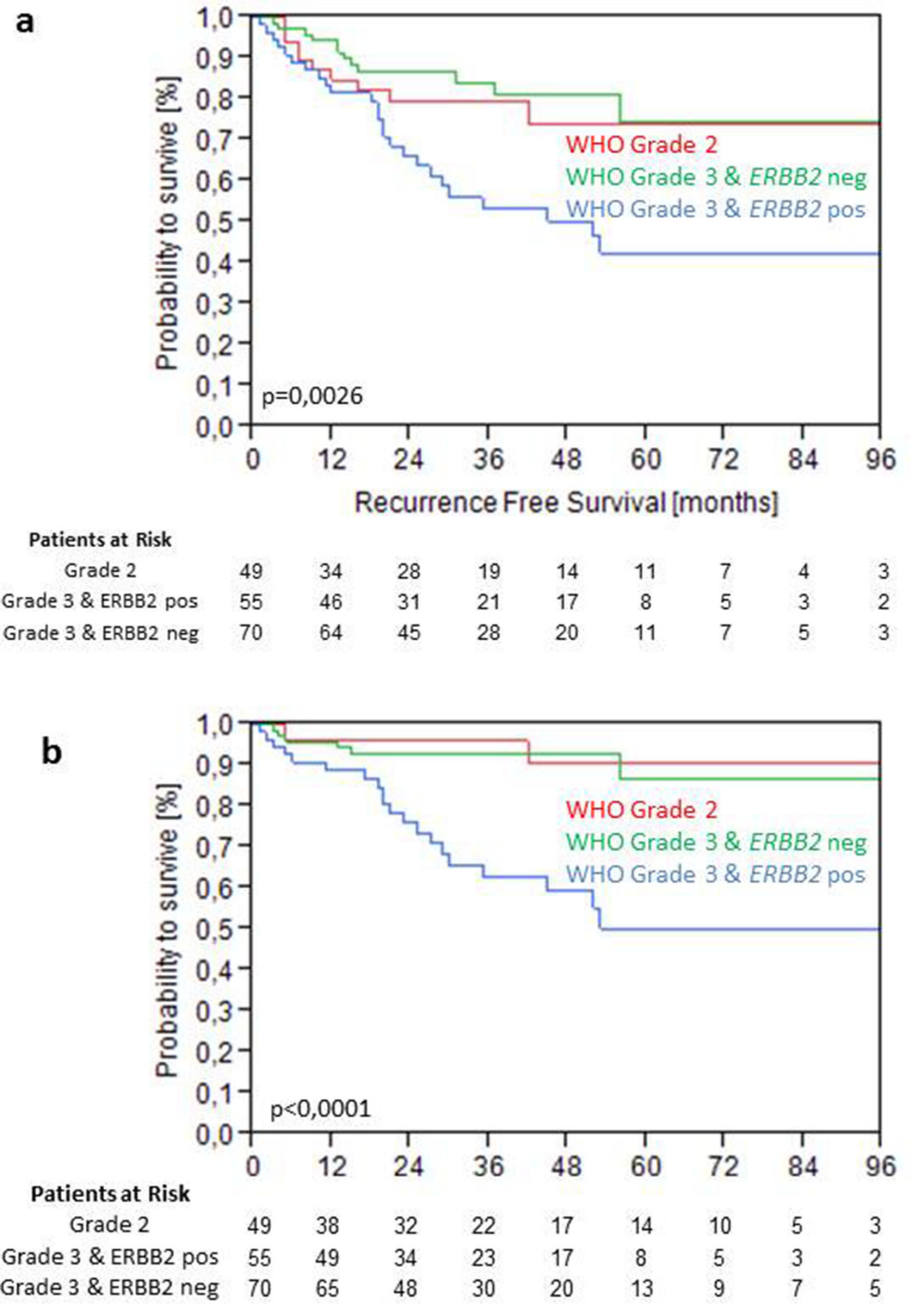
Kaplan-Meier analysis for RFS **(a)** and PFS **(b)** stratified by WHO Grade 1973 and intermediate ERBB2 mRNA levels at 40.1 in the validation cohort.

Higher levels of ERBB2 mRNA were not significant for PFS as continuous variable in the total cohort. However, elevated expression of HER2 above 40.1 was highly significant for PFS (p=0.0025) and showed a trend towards significance for RFS (p=0.0587) (Figure 4). For PFS elevated ERBB2 mRNA expression significantly discriminated a high risk group with only 60% PFS after 5 years from a low risk group with 90% PFS after 5 year follow-up within WHO 1973 Grade 3 tumors (p<0.001) (Figure 4). Grade 3 tumors with low ERBB2 mRNA expression (<40.1) showed the same recurrence and progression rates as Grade 2 tumors (Figure 5).

## DISCUSSION

Reliable risk assessment is critical for individualized treatment decisions for stage pT1 bladder cancer patients [2]. By now stratification is done using conventional clinical and pathologic features like tumor diameter, focality and pathologic grading [2].

In the present study we used an established IVD kit to determine mRNA-expression of ERBB2, ESR1, PGR and MKI67. We could show that in NMIBC similar subtypes and hormone receptor expression like in breast cancer could be found. Breast cancer can be divided into subtypes which show differences in the survival rates, risk of recurrence and response to systemic therapy [12]. Compared to breast cancer, in the present study we could show similar frequencies for Luminal-B-like tumors [13]. Luminal-A and HER2-positive subtypes occurred less frequent than in breast cancer, whereas Triple negative subtypes occurred more frequent [13]. These results show that there is hormone receptor expression in NMIBC, with an organ-dependent expression pattern. For MIBC luminal-like, basal and p-53-like subtypes mimicking those in breast cancer could be identified [14]. Luminal subtypes show expression of ESR1 and HER2 [14]. HER2 amplification can be found in 7% of MIBCs, which is in line with our results for pT1 NMIBC (12.9%) [5]. For MIBC a different response to neoadjuvant cisplatin-based chemotherapy could be found, like seen in breast cancer. Luminal-A tumors, especially p53-like subtypes, show the worst response rate to chemotherapy [6]. This indicates to other potential therapeutic targets in these tumors like HER2- or the ESR1-receptor. To date this is the only study which describes hormone receptor expression and subtype stratification in solely pT1 NMIBC.

Subgroup classification (Table 2) was significant for prediction of progression and independent from current clinical routine parameters, with Luminal-B tumors showing the worst RFS and PFS. As Luminal-B like tumors are mainly ESR1 positive, the activation of the ESR1-pathway seems to be the driving force for recurrence and progression. High estrogen levels are commonly seen as being protective for the development of bladder cancer [15]. For MIBC ESR1 positivity is seen as a luminal marker [14]. Risk stratification as done in the present study can be predictive and also reveal therapeutic targets. Selective estrogen receptor modulators (SERMs) are used for anti-cancer treatment of breast cancer [16]. In an urothelial carcinoma cell line it could be shown that SERMs can inhibit tumor cell proliferation [17]. In line with our results for NMIBC, hormone receptor positive tumors with low proliferation show the best survival rates in breast cancer with HER2 positive and triple negative tumors exhibiting worse survival. However, hormone receptor positive tumors, with intermediate expression of HER2 and high proliferation rate have the worst prognosis, which is different from the situation in breast cancer [18].

For predicting prognosis and recurrence of NMIBC risk calculators including easy to assess clinical and pathological features have been established [19, 20]. Further molecular and pathologic markers to improve risk stratification in NMIBC have been evaluated, i.e. the extension of invasive growth in pT1 disease [21, 22], FGFR3 and KI-67 for recurrence [21, 23] or cathepsin E and survivin for progression [24]. In contrast to these results, subtyping in the present study can provide an objective and easy to assess risk stratification for both recurrence and progression. The molecular subtypes could predict those and could add to the tumor differentiation and thus can be an additional objective risk calculator. Furthermore, ESR1, PGR and HER2 display potential drug targets.

High levels of HER2 expression were statistically significant for PFS in the cohort of pT1 bladder cancer and could determine worse PFS within the group of pT1G3 tumors. These high risk tumors represent a challenging entity for the treating urologist with either bladder preserving therapy with a maintenance BCG instillation therapy or radical cystectomy [2]. For this subentity of pT1G3 tumors, early cystectomy is associated with an improved long-term cancer-specific survival [25]. To date it is difficult to identify patients at risk for progression, who will benefit from an early cystectomy. Clinical factors like age or tumor diameter could be identified as risk factors [26]. In a small series of patients with pT1G3 bladder cancer under treatment with BCG, of 7 IHC markers tested, none with predictive value for recurrence or progression could be found [27]. In an IHC analysis in pT1G3 NMIBC, HER2 expression was not associated to recurrence or progression [28]. The weakness of IHC based studies is a lack of standardization and of objectiveness. The results for HER2 mRNA expression as well as the other markers in the present study are reproducible and objective and can easily be standardized [11]. Thus a high HER2 expression can help to identify patients who will benefit from early cystectomy. Furthermore HER2 also represents a target for a targeted therapy with drugs used in breast cancer today. These patients with high expression of HER2 may undergo specific anti-HER2 treatment instead of early cystectomy.

The major weakness of the present study is its retrospective nature with data from a single-center. To confirm and verify the results a validation in a multi-center study will be necessary. The lack of predictive value for cancer-specific survival may be due to few events and the short median follow-up.

To conclude, hormone receptor expression of ESR1, PGR and HER2 can be detected in NMIBC and has prognostic value. This study proved for the first time, that stage pT1 NMIBC can be classified almost identically to breast cancer and expresses prognostic relevant therapeutic targets, which are the basis for efficient breast cancer treatment.

## MATERIALS AND METHODS

### Study population

Of 302 patients (finding cohort n=100, evaluation cohort n=202) we could include 255 patients with stage pT1 NMIBC (78.8% male) at initial diagnosis who underwent transurethral resection of the bladder (TURB). Histopathological parameters of all cases, including grading according to WHO 1973 and WHO 2004 classification were assessed by a pathologist specialized in uropathology (A.H.). All patients underwent reresection and were treated according to an organ preserving approach. Progress was defined as progression to muscle-invasive disease. The study was conducted after approval of the local ethics committee. Informed consent was obtained.

### Isolation of tumor RNA

For RNA extraction from FFPE tissue, a single 10 μm curl was processed according to a commercially available bead-based extraction method (RNXtract^®^ kit; BioNTech Diagnostics GmbH, Mainz, Germany). RNA was eluted with 100 μl elution buffer and RNA eluates were then stored at −80°C until use.

### Gene expression by RT-qPCR

MammaTyper^®^ is a molecular *in vitro* diagnostic tool for the assessment of the gene expression levels of the four cancer biomarkers that are required for the clinical management of breast cancer patients in daily routine. Instead of using IHC to assess protein expression of HER2, ESR1, PR, and Ki-67, with MammaTyper^®^, it is possible to measure the mRNA transcripts of the corresponding genes (ERBB2, ESR1, PGR, and MKI67), doing so by using routine FFPE material and by achieving accurate, reproducible and objective results as outlined in Laible et al [11].

The mRNA expression levels of ERBB2, ESR1, PGR, and MKI67 as well as of two reference genes (REF), namely B2M and CALM2, were determined by RT-qPCR, which involves reverse transcription of RNA and subsequent amplification of cDNA executed successively as a 1-step reaction. In MammaTyper^®^, the 6 assays (assay = primer pair and probe specific for the respective target sequence) are duplexed into three assay mixes, each using a pair of hydrolysis probes labeled with different fluorophores for separate detection of the duplexed assays.

Each patient sample or control was analyzed with each assay mix in triplicates. The experiments were run on a Light Cycler LC480 II (Roche, CH) according to the following protocol: 5 min at 50°C, 20 sec at 95°C followed by 40 cycles of 15 sec at 95°C and 60 sec at 60°C and according to MammaTyper^®^ instructions for use 140603-90020-EU Rev 2.0.

Forty amplification cycles were applied and the cycle quantification threshold (Cq) values of four markers and the two REF genes for each sample (S) were estimated as the median of the triplicate measurements. These were then normalized against the mean expression of the REF genes and set off against a synthetic *in vitro* transcribed RNA calibrator included in the MammaTyper kit (PC), to correct for inter-run variations (ΔΔCq method). The final values were generated by subtracting ΔΔCq from the total number of cycles to ensure that normalized gene expression obtained by the test is proportional to the corresponding mRNA expression levels. The various calculation steps are summarized in the following formula (exemplary for MKI67):

40-ΔΔCq(MKI67)S = 40-((Cq[MKI67]S – meanCq[REF]S) – (Cq[MKI67]pc – meanCq[REF]pc))

### Molecular subtyping

Similar to the situation in breast cancer the main molecular classes of bladder cancer shall be distinguished to date depending on the expression of HER2, ER, PR and Ki-67. Due to limited number of samples in this cohort, a simplified subtype classification was used here (Table 2).

Predefined Cut-Off values used in the validation series were as follows: *ERBB2* ≥ 41.10, *ESR1* ≥ 36.12, *PGR* ≥ 32.8, *MKI6*7 ≥36.52.

### Statistical methods

Statistical analysis has been performed using SPSS version 23 and JMP 9.0.0. Cut-Off definitions were done in a finding cohort of 100 T1 NMIBC by Partitioning tests and Youden Index analysis. Molecular subtyping has been performed by predefined algorithm related to the MammaTyper^®^ classification and with adopted Cut-Offs for ESR1, PGR, ERBB2 and MKI67. After biostatistical survival analysis of the finding cohort and finalization of cut-off and subtype definition the molecular data of a validation cohort of 202 stage pT1 NMIBC had been connected with clinical follow-up information. After initial validation data had been pooled for exploratory analysis and outcome related cut-off optimization for ERBB2. Statistical analysis including Kaplan-Meier survival analysis, multivariate cox regression and partitioning testing were performed with JMP SAS (SAS Institute, Cary, NC, USA) and Graph Pad Prism software (Version 5.04; Graph Pad Software Inc., La Jolla, CA, USA).

## Abbreviations

ESR1/ER: estrogen receptor alpha
ERBB2/HER2: estrogen receptor beta 2/glutamyl-tRNA(Gln) amidotransferase subunit HER2
Cq: quantification cycle
CSS: cancer-specific survival
FFPE: formaline fixed paraffin embedded
GOI: gene of Interest
HR: hazard ratio
mRNA: messenger RiboNucleinacid
MKI67/Ki67: marker of proliferation Ki-67
OS: overall survival
PGR/PgR: progesterone receptor
PFS: progression-free survival
REF: reference gene
RFS: recurrence-free survival
RT-qPCR: reverse transcription quantitative real time polymerase chain reaction

## References

[1] Jemal A, Bray F, Center MM, Ferlay J, Ward E, Forman D (2011). Global cancer statistics. CA Cancer J Clin.

[2] Babjuk M, Burger M, Zigeuner R, Shariat SF, van Rhijn BW, Comperat E, Sylvester RJ, Kaasinen E, Bohle A, Palou Redorta J (2013). EAU guidelines on non-muscle-invasive urothelial carcinoma of the bladder: update 2013. Eur Urol.

[3] Shahin O, Thalmann GN, Rentsch C, Mazzucchelli L, Studer UE (2003). A retrospective analysis of 153 patients treated with or without intravesical bacillus Calmette-Guerin for primary stage T1 grade 3 bladder cancer: recurrence, progression and survival. J Urol.

[4] Hong YM, Loughlin KR (2008). Economic impact of tumor markers in bladder cancer surveillance. Urology.

[5] Cancer Genome Atlas Research Network (2014). Comprehensive molecular characterization of urothelial bladder carcinoma. Nature.

[6] Choi W, Porter S, Kim S, Willis D, Plimack ER, Hoffman-Censits J, Roth B, Cheng T, Tran M, Lee IL, Melquist J, Bondaruk J, Majewski T (2014). Identification of distinct basal and luminal subtypes of muscle-invasive bladder cancer with different sensitivities to frontline chemotherapy. Cancer Cell.

[7] Damrauer JS, Hoadley KA, Chism DD, Fan C, Tiganelli CJ, Wobker SE, Yeh JJ, Milowsky MI, Iyer G, Parker JS, Kim WY (2014). Intrinsic subtypes of high-grade bladder cancer reflect the hallmarks of breast cancer biology. Proc Natl Acad Sci U S A.

[8] Patschan O, Sjodahl G, Chebil G, Lovgren K, Lauss M, Gudjonsson S, Kollberg P, Eriksson P, Aine M, Mansson W, Fernö M, Liedberg F, Höglund M (2015). A molecular pathologic framework for risk stratification of stage T1 urothelial carcinoma. Eur Urol.

[9] Sjodahl G, Lauss M, Lovgren K, Chebil G, Gudjonsson S, Veerla S, Patschan O, Aine M, Ferno M, Ringner M, Månsson W, Liedberg F, Lindgren D (2012). A molecular taxonomy for urothelial carcinoma. Clin Cancer Res.

[10] Rebouissou S, Bernard-Pierrot I, de Reynies A, Lepage ML, Krucker C, Chapeaublanc E, Herault A, Kamoun A, Caillault A, Letouze E, Elarouci N, Neuzillet Y, Denoux Y (2014). EGFR as a potential therapeutic target for a subset of muscle-invasive bladder cancers presenting a basal-like phenotype. Sci Transl Med.

[11] Laible M, Schlombs K, Kaiser K, Veltrup E, Herlein S, Lakis S, Stöhr R, Eidt S, Hartmann A, Wirtz RM, Sahin U (2016). Technical validation of an RT-qPCR in vitro diagnostic test system for the determination of breast cancer molecular subtypes by quantification of ERBB2, ESR1, PGR and MKI67 mRNA levels from formalin-fixed paraffin-embedded breast tumor specimens. BMC Cancer.

[12] Guiu S, Michiels S, André F, Cortes J, Denkert C, Di Leo A, Hennessy BT, Sorlie T, Sotiriou C, Turner N, Van de Vijver M, Viale G, Loi S (2012). Molecular subclasses of breast cancer: how do we define them? The IMPAKT 2012 Working Group Statement. Ann Oncol.

[13] Koutras A, Kalogeras KT, Wirtz RM, Alexopoulou Z, Bobos M, Zagouri F, Veltrup E, Timotheadou E, Gogas H, Pentheroudakis G, Pisanidis N, Magkou C, Christodoulou C (2015). Evaluation of the prognostic significance of HER family mRNA expression in high-risk early breast cancer: a Hellenic Cooperative Oncology Group (HeCOG) validation study. J Transl Med.

[14] Choi W, Czerniak B, Ochoa A, Su X, Siefker-Radtke A, Dinney C, McConkey DJ (2014). Intrinsic basal and luminal subtypes of muscle-invasive bladder cancer. Nat Rev Urol.

[15] Hsu I, Vitkus S, Da J (2013). Role of estrogen receptors in bladder cancer development. Nat Rev Urol.

[16] Lumachi F, Santeufemia DA, Basso SM (2015). Current medical treatment of estrogen receptor-positive breast cancer. World J Biol Chem.

[17] Hoffman KL, Lerner SP, Smith CL (2013). Raloxifene inhibits growth of RT4 urothelial carcinoma cells via estrogen receptor-dependent induction of apoptosis and inhibition of proliferation. Horm Cancer.

[18] Bulut N, Altundag K Does estrogen receptor determination affect prognosis in early stage breast cancers?. Int J Clin Exp Med.

[19] Sylvester RJ, van der Meijden A, Oosterlinck W, Witjes JA, Bouffioux C, Denis L, Newling DW, Kurth K (2006). Predicting recurrence and progression in individual patients with stage Ta, T1 bladder cancer using EORTC risk tables: a combined analysis of 2596 patients from seven EORTC trials. Eur Urol.

[20] Fernandez-Gomez J, Madero R, Solsona E, Unda M, Martinez-Piñeiro L, Gonzalez M, Portillo J, Ojea A, Pertusa C, Rodriguez-Molina J, Camacho JE, Rabadan M, Astobieta A (2009). Predicting non-muscle invasive bladder cancer recurrence and progression in patients treated with bacillus Calmette-Guerin: the CUETO scoring model. J Urol.

[21] van Rhijn BW, Liu L, Vis AN, Bostrom PJ, Zuiverloon TC, Fleshner NE, van der Aa MN, Alkhateeb SS, Bangma CH, Jewett MA, Zwarthoff EC, Bapat B, van der Kwast TH (2012). Prognostic value of molecular markers, sub-stage and European Organisation for the Research and Treatment of Cancer risk scores in primary T1 bladder cancer. BJU Int.

[22] Bertz S, Denzinger S, Otto W, Wieland WF, Stoehr R, Hofstaedter F, Hartmann A (2011). Substaging by estimating the size of invasive tumour can improve risk stratification in pT1 urothelial bladder cancer-evaluation of a large hospital-based single-centre series. Histopathology.

[23] Bertz S, Otto W, Denzinger S, Wieland WF, Burger M, Stöhr R, Link S, Hofstädter F, Hartmann A (2014). Combination of CK20 and Ki-67 immunostaining analysis predicts recurrence, progression, and cancer-specific survival in pT1 urothelial bladder cancer. Eur Urol.

[24] Fristrup N, Ulhøi B, Birkenkamp-Demtröder K, Mansilla F, Sanchez-Carbayo M, Segersten U, Malmström PU, Hartmann A, Palou J, Alvarez-Múgica M, Zieger K, Borre M, Ørntoft TF (2012). Cathepsin E, maspin, pik1, and survivin are promising prognostic protein markers for progression in non-muscle invasive bladder cancer. Am J Pathol.

[25] Denzinger S, Fritsche HM, Otto W, Blana A, Wieland WF, Burger M (2008). Early versus deferred cystectomy for initial high-risk pT1G3 urothelial carcinoma of the bladder: do risk factors define feasibility of bladder-sparing approach?. Eur Urol.

[26] Gontero P, Sylvester R, Pisano F, Joniau S, Vander Eeckt K, Serretta V, Larré S, Di Stasi S, Van Rhijn B, Witjes AJ, Grotenhuis AJ, Kiemeney LA, Colombo R (2015). Prognostic factors and risk groups in T1G3 non-muscle-invasive bladder cancer patients initially treated with Bacillus Calmette-Guérin: results of a retrospective multicenter study of 2451 patients. Eur Urol.

[27] Park J, Song C, Shin E, Hong JH, Kim CS, Ahn H (2013). Do molecular biomarkers have prognostic value in primary T1G3 bladder cancer treated with bacillus Calmette-Guerin intravesical therapy?. Urol Oncol.

[28] Bongiovanni L, Arena V, Vecchio FM, Racioppi M, Bassi P, Pierconti F (2013). HER-2 immunohistochemical expression as prognostic marker in high-grade T1 bladder cancer (T1G3). Arch Ital Urol Androl.

